# Mechanistic redundancy and hierarchy of aging mechanisms: implications for strategies to extend healthspan and biomarker integration

**DOI:** 10.3389/fragi.2026.1776669

**Published:** 2026-06-29

**Authors:** Maria Shvedova

**Affiliations:** BUMC Center for Aging Research, Division of Plastic and Reconstructive Surgery, Department of Surgery, Boston University School of Medicine, Boston, MA, United States

**Keywords:** aging, biomarkers, DNA damage, estrogen, menopause, oxidative stress, preventive interventions, telomere attrition

## Abstract

Aging involves a network of interrelated biological processes that differ in causality and impact. This review proposes a hierarchical framework of primary, secondary, and tertiary drivers of human aging while emphasizing the extensive feedback loops and mechanistic redundancy. Primary mechanisms include molecular damage, particularly genomic and mitochondrial DNA damage, and mutation accumulation that cumulatively result in genomic instability, and telomere attrition, which represents a separate primary driver. Although rarely recognized as an independent aging mechanism, female sex hormone decline, particularly the abrupt loss of sex steroid signaling at menopause and earlier perimenopausal changes, may constitute a primary sex-specific driver of human aging as an evolved process that amplifies molecular and physiological deterioration. Mechanisms acting as both primary and secondary include damage to molecules other than DNA including protein damage with loss of proteostasis and lipid damage, which may arise directly from molecular insults or emerge as downstream consequences of DNA damage and other primary mechanisms, while also feeding back to accelerate upstream deterioration. Secondary mechanisms comprise cellular senescence, impaired macroautophagy, deregulated nutrient sensing, epigenetic alterations, mitochondrial dysfunction, and altered intercellular communication. These processes emerge downstream of initial damage and further reciprocally reinforce it. Tertiary mechanisms of aging comprise stem cell exhaustion, chronic inflammation, and dysbiosis, which represent the system-level deterioration exacerbating molecular dysfunction. This hierarchical network-based model suggests that many hallmarks of aging may represent manifestations of redundant upstream molecular insults. This review focuses on primary mechanisms as the causative drivers of aging and proposes that the strategy to extend healthspan may require preventive approaches targeting distinct redundant primary mechanisms. Complex preventive interventions that simultaneously reduce molecular damage, slow telomere attrition, and compensate for estrogen depletion may delay the initiation and amplification of secondary and tertiary aging mechanisms. This framework supports coordinated multi-target strategies for healthspan extension and underscores the need for validated biomarkers that reflect these upstream processes as part of an integrated preventive approach.

## 1 Introduction

The increase of average age worldwide poses new socioeconomic and healthcare challenges of unprecedented scale (Bluethmann et al., 2016; Nikolich-Žugich et al., 2016), representing a significant unmet need in strategies to increase healthspan. The development of such strategies requires a framework distinguishing primary drivers of aging to allow development of focused preventive interventions.

According to common definition, aging is characterized by the progressive functional decline and increased mortality risk shared by nearly all metazoans and associated with molecular and cellular changes (Schumacher et al., 2008). The root cause of the aging process has been debated, with the array of theories encompassing time-dependent molecular damage accumulation and evolutionary aging theories including seminal works in the field such as antagonistic pleiotropy, disposable soma, and mutation accumulation due to decreased natural selection against deleterious mutations with phenotypical effects manifesting in post-reproductive period (Ljubuncic and Reznick, 2009; Medawar, 1953; Williams, 1957). It has even been recently demonstrated that researchers working on aging today still do not agree on the mechanisms underlying the process (Gladyshev et al., 2024) while competing theories and frameworks proliferate rather than converge into a unified conceptual model. It becomes increasingly clear, however, that different theories and proposed mechanisms are not mutually exclusive since aging is a multifactorial process involving multiple overlapping mechanisms which reinforce each other (da Costa et al., 2016). Gradual accumulation of damage occurs across molecular systems including the genome, proteome, lipidome, and epigenome due to imperfectness of molecular repair systems, which represents a major driver of aging (Schumacher et al., 2008; Hayflick, 2007; Gladyshev et al., 2021; Gladyshev, 2013; Yousefzadeh et al., 2021; Gensler and Bernstein, 1981; Schumacher et al., 2021; Hoeijmakers, 2009). This damage arises from both endogenous stressors and environmental exposures, with oxidative stress emerging as a central generator of molecular damage across aging tissues (Shigenaga et al., 1994; Barja, 2002; J et al., 2023; Martien and Abbadie, 2007; Barja and Herrero, 2000).

DNA damage has been hypothesized to represent the earliest evolutionary cause of aging which has been present from the origin of life and which, with increasing complexity of life (Yousefzadeh et al., 2021; Schumacher et al., 2021), later was joined by additional evolutionarily developed causes which act relatively independently of molecular damage *per se* and facilitate aging-related decline, such as telomere attrition, and serving as double-edged swords–decreasing cancer risk earlier in life but advancing aging (Schumacher et al., 2021; de Magalhães, 2025a). Hormonal decline is another change occurring with aging (Hertoghe, 2005); which at least in females with abrupt menopausal changes represents an evolutionary outcome (Blagosklonny, 2010; Xing Y. et al., 2023; Jaschke et al., 2021; Finch, 1994; Olson, 1987) with deleterious health consequences (Ossewaarde et al., 2005; Zhu et al., 2019; Huang et al., 2023; Xing Z. et al., 2023).

**FIGURE 1:**
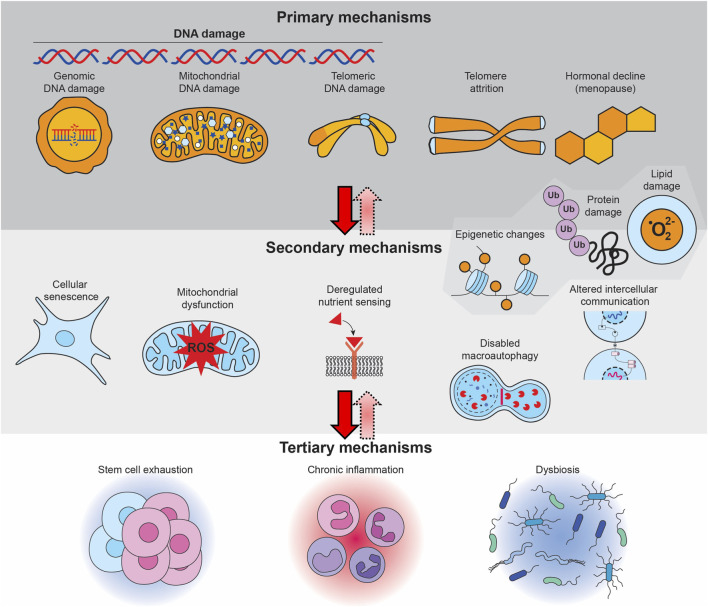
Hierarchical organization of aging mechanisms and feedback structure. Aging is initiated by redundant primary mechanisms, including genomic, mitochondrial, and telomeric DNA damage, telomere attrition, and sex-hormone decline, which destabilize cellular homeostasis. Damage to proteins and lipids can act both as primary insults when affecting long-lived or aggregation-prone molecules and as secondary consequences of genomic instability, weakened maintenance systems, and increasing oxidative stress. These upstream pressures give rise to secondary mechanisms, including cellular senescence, mitochondrial dysfunction, deregulated nutrient sensing, impaired autophagy, altered intercellular communication, and predominantly secondary epigenetic alterations, which also retain limited primary capacity through direct chromatin and transcriptional disruption. Accumulation of primary and secondary dysfunction ultimately produces tertiary outcomes, such as stem cell exhaustion, chronic inflammation, and dysbiosis, reflecting late-stage tissue and organismal decline.

These mechanisms–molecular damage resulting from intrinsic and extrinsic insults coupled with imperfectness of repair mechanisms (Gladyshev, 2013; Olson, 1987) (in particular, DNA damage and mutation accumulation, which, in turn, result in genomic instability (Schumacher et al., 2021; Vijg, 2021; Vijg, 2014)), telomere attrition (Schellnegger et al., 2024; West et al., 2019), and hormonal decline (at least in females (Finch, 1994)) – can be conceptualized as primary drivers of aging (Figure 1). Damage to molecules other than DNA, such as proteins and lipids can function as a primary mechanism when it affects long-lived or aggregation-prone macromolecules (Yousefzadeh et al., 2021; Schumacher et al., 2021). However, it also arises secondarily from genomic instability, as mutations and transcriptional errors generate dysfunctional proteins and weaken maintenance systems. Once established, proteome and lipidome injury can feed back to worsen genomic instability, for example, by impairing DNA repair, creating reinforcing loops. The primary drivers of human aging are redundant since they develop via different pathways and, therefore, targeting one pathway alone is insufficient, as others will still drive aging (López-Otín et al., 2013; López-Otín et al., 2023; Aunan et al., 2016). These findings emphasize that aging may emerge from partially overlapping upstream processes, reinforcing the concept of mechanistic redundancy within the proposed framework.

Secondary mechanisms/hallmarks, which ensue from the primary mechanisms either as a direct consequence or as adaptive responses (Coler-Reilly et al., 2025), include cellular senescence (Herbig et al., 2004; Reaper et al., 2004; Lou and Chen, 2006), epigenetic alterations (Sinclair and Oberdoerffer, 2009), deregulated nutrient sensing (Schumacher et al., 2008; Schumacher et al., 2021), mitochondrial dysfunction (Fang et al., 2016; Passos et al., 2007; Zole and Ranka, 2018; Cline, 2012), disabled macroautophagy, and altered intercellular communication (Aunan et al., 2016; da Silva and Schumacher, 2021). It is important to note that the distinction of some of the mechanisms as secondary, such as epigenetic alterations, is not clear-cut as they may partially constitute primary aging mechanisms; however, they mostly emerge secondary to genomic instability. Tertiary hallmarks consist in dysfunction of higher cellular organization and include stem cell exhaustion (Fathi et al., 2019; Zhong et al., 2024; Schuler and Rübe, 2013; Rübe et al., 2011; Rossi et al., 2007; Chinvattanachot et al., 2024), chronic inflammation, and dysbiosis triggered by the primary and secondary mechanisms (López-Otín et al., 2023).

In this hypothetical framework, mechanisms are classified as “primary” when they represent initiating or upstream processes that can independently give rise to multiple downstream aging hallmarks and whose experimental modulation alters aging trajectories across systems. “Secondary” mechanisms are defined as processes that arise in response to primary damage or dysregulation and that amplify or propagate aging phenotypes. “Tertiary” mechanisms refer to higher-level tissue or systemic manifestations that emerge from cumulative upstream dysfunction. This conceptual classification reflects relative causal positioning within a network that includes substantial feedback and context dependence.

While basic aging mechanisms are highly conserved in most species (Pitt and Kaeberlein, 2015), significant interspecies divergence still exists. For example, telomere attrition, while eliciting replicative senescence in human cells, is less relevant to the natural aging in many inbred laboratory mouse strains, which have constitutively high telomerase expression and long telomeres (Gomes et al., 2011). This review focuses on the primary mechanisms relevant to human aging, and provides some examples from other species in the context of comparative biology or when human findings are incomplete. While the established hallmarks of aging framework offers a valuable structure (López-Otín et al., 2013; López-Otín et al., 2023; Aunan et al., 2016), this review proposes hypothesis-driven hierarchical framework in which aging mechanisms are organized according to their likely causal position and emphasizes causal redundancy which suggests the need for development of multi-target preventive strategies and biomarker integration, while recognizing that substantial feedback, interdependence, and context-dependent variation exist.

## 2 Primary mechanisms

The primary mechanisms initiate a cascade of dysfunction, setting the stage for secondary and tertiary processes that amplify and diversify aging phenotypes. It has been hypothesized that the generation and clearance/dilution of the damage operated ever since the first cellular organisms and are deeply rooted in metabolism, and that aging results from imperfectness of repair processes (Gladyshev, 2013), suggesting that the damage accumulates not because it is unrepairable in principle, but because the cost of its repair outweigh the evolutionary benefit (Olson, 1987). However, no single factor can account for the entirety of the aging process (Vijg, 2021), and later damage accumulation was joined by telomere attrition as additional cause driving cellular senescence in humans (Gomes et al., 2011). While most hormonal alterations occurring with aging can be considered as a process resulting from the accumulated damage, telomere attrition, and downstream changes, limited ovarian reserve in females can be considered as an independent factor reducing healthspan and accelerating aging-related diseases (Ossewaarde et al., 2005; Asllanaj et al., 2019). This section outlines the causal role of these mechanisms as redundant drivers of the aging process.

### 2.1 DNA damage and mutation accumulation resulting in genomic instability

#### 2.1.1 Evolutionary perspective on DNA damage as the core driver of aging

Molecular damage has been hypothesized to be the primary driving force of aging both at the individual and evolutionary levels (Gladyshev et al., 2021; Vijg, 2021; Kirkwood and Austad, 2000; Meyer et al., 2025). Indeed, the origin of genome maintenance was among the first selected traits, possibly tracking back to the first replicators (Vijg, 2014; Bernstein et al., 1987; Aravind et al., 1999). DNA damage, in particular has been hypothesized to be the primary aging-driving force acting upon cells since the origin of life, and DNA represents irreplaceable template which, when modified by mutations or damaged without repair, causes irreversible changes in cellular function (Vijg, 2021). This notion is supported by the discovery that repair and antioxidant enzymes were among the earliest evolutionary adaptations, which likely strengthened around 2.5 billion years ago during the Great Oxidation Event, when rising atmospheric oxygen levels increased oxidative stress (Burrows, 2009). The rate of occurrence of DNA damage, the rate of DNA repair (Hart et al., 1979), the degree of cellular redundancy, and the extent of exposure to stress have been proposed as factors which determine maximum longevity (Gensler and Bernstein, 1981). While specific mechanisms of DNA damage responses significantly vary across living organisms, general principles are conserved from bacteria and archaea to humans (Williams and Schumacher, 2016).

From the comparative biology standpoint, the DNA repair mechanisms and antioxidant defenses have been demonstrated to be more robust in long-living compared to those in short-living species (Holliday, 2004; Kirkwood, 2008; Austad, 2010; Cutler, 1991a; Cutler, 1991b), while ROS production is characterized by the opposite trend (Lombard et al., 2005; Perez-Campo et al., 1998). It has been proposed that human aging is largely driven by DNA damage arising from oxidative stress and imperfect repair, and that the long-lived species, including humans, evolved enhanced antioxidative defenses and genomic stability under strong selective pressure (Strehler, 1986). In accordance with damage-accumulation theory of aging, comparative biology approach identified that proteins involved in DNA damage repair and proteasome–ubiquitin system exhibit longevity-specific selection patterns (Li and De Magalhães, 2011). Differences in genome instability across species may reflect variations in genome robustness, with simpler regulatory networks in species such as *Drosophila* conveying tolerance to higher mutation loads despite less efficient genome maintenance (Vijg and Suh, 2013). In humans, variants of several genome maintenance genes, such as p53, *MLH1*, *TERT/TERC*, *FOXO3A*, and *XPD*, have been associated with increased longevity (Vijg and Suh, 2013).

The evolutionary significance of DNA damage is further highlighted by the hypothesis that sexual reproduction, at least in part, has evolved as a mean to mitigate DNA damage irreparable by regular DNA repair mechanisms through recombination of germline DNA (Gensler and Bernstein, 1981; Avise, 1993). Even in phage and bacterial systems recombination processes has been demonstrated to be effective at repairing DNA lesions (Gensler and Bernstein, 1981). In line with this hypothesis, somatic mutation accumulation has been proposed as one of the explanations why mammalian males have shorter lifespan than females - since mammalian males are the heterogametic sex lacking second X chromosome (Wolf, 2021a). However, when females are the heterogametic sex (such as birds), male birds appear to have comparable or longer lifespans than female birds (Xirocostas et al., 2020). Homogametic sex lives on average 17.6% longer than heterogametic sex (Xirocostas et al., 2020). This trend is consistent with greater expression of recessive deleterious mutations in the heterogametic sex, owing to the lack of a second sex chromosome, alongside additional sex-chromosome dosage effects such as biallelic expression of tumor suppressor genes escaping X-inactivation (Dunford et al., 2016).

#### 2.1.2 Genomic DNA damage

Nuclear DNA is particularly vulnerable to aging-related changes because, unlike most other cellular components that can be continuously replaced, it must persist throughout the cell’s lifespan, and the nuclear genome exists in only two to four copies per cell, making it more vulnerable to damage (Lombard et al., 2005; Freitas and De Magalhães, 2011). DNA damage is caused by free radicals, extrinsic radiation, environmental mutagens, as well as errors during cell replication (Gladyshev et al., 2021; Vijg and Suh, 2013), and it has been postulated that accumulation of damage and mutations further contributes to decrease in DNA repair capacity (for example, due to damage to the genes encoding DNA repair enzymes) forming vicious cycle leading to genomic instability (Gorbunova et al., 2007). DNA damage and mutations accumulate with aging in multiple mammalian organs and tissues including brain, skeletal muscles, heart, spleen, intestine, ovaries, blood, and retina (Gensler and Bernstein, 1981; Vijg and Suh, 2013; Hart and Turturro, 1981; Lieber and Karanjawala, 2004).

DNA damage is hypothesized to be a primary causal factor in the aging process, in part through blocking transcription and induction of cellular senescence (Vijg, 2021). Age-related DNA damage alters chromatin structure in part via p53-dependent suppression of core histone gene expression, which increases transcription start sites accessibility and disrupts transcriptional integrity. These chromatin changes lead to widespread RNA polymerase II stalling, cryptic transcription initiation within gene bodies, and accelerated elongation speed, collectively compromising RNA fidelity, splicing accuracy, and overall transcriptional homeostasis during aging (Papantonis et al., 2024).

One of the most common oxidative DNA modifications, and therefore most widely used marker of oxidative stress, is 8-oxoguanine (8-OHdG) (Jung et al., 2024; Gao et al., 2019) - has been demonstrated to accumulate with age (Lombard et al., 2005; Hamilton et al., 2001). Another marker of DNA damage - DNA damage foci of cultured human fibroblasts are also significantly associated with the chronological donor age (Waaijer et al., 2016). The role of DNA damage in driving cellular senescence is underscored by findings from both cultured cells and mouse liver, where polyploidy allowed cells to evade cell cycle arrest and decrease expression of senescence markers despite accumulation of DNA damage, highlighting how additional genomic content may buffer the functional consequences of DNA damage (Hayashi et al., 2024).

Transposable element activity increases with aging across species, including *Drosophila*, mice, and humans, largely due to age-associated erosion of chromatin-based silencing mechanisms such as DNA methylation, histone modifications, and RNA interference (Merenciano et al., 2025). Age-related transcriptional errors, including increased intron retention and transcriptional readthrough, increase with age and further exacerbate transposable element activation (Merenciano et al., 2025). Reactivation of these elements directly contributes to genomic instability through insertional mutagenesis and promotes chronic inflammation, reinforcing DNA damage accumulation during aging (Merenciano et al., 2025).

#### 2.1.3 Mutation accumulation

Age-associated mutation accumulation arises in part from imperfect resolution of DNA damage, as repair mechanisms fail to fully or accurately restore genomic integrity over time (Moskalev et al., 2013). The accumulation of mutations in essential genes may represent an important mechanism through which imperfect DNA repair contributes to aging (Lombard et al., 2005). Mutation accumulation in virtually every tissue during the aging process has been confirmed, and is postulated to contribute to transcriptional noise which, in turn, can cause cellular fate drift and defects in cell signaling, as well as stoichiometric imbalance in the assembly of multimolecular complexes, which in turn causes protein aggregation occurring with aging, while clonal development of mutations contribute to increased disease risk (Vijg, 2021).

The rate of mutation accumulation is most rapid during early and late life (Podolskiy et al., 2016), consistent with high proliferative rate early in life and genomic instability with decrease in DNA repair capacity later in life. Consistent with disposable soma theory, the mutation rate in somatic cells is one to two orders of magnitude greater than in germ cells (Vijg, 2021; Milholland et al., 2017).

From a comparative biology perspective, somatic mutation rates per year inversely correlate with species lifespan, indicating that long-lived species accumulate mutations more slowly in adult tissues, likely reflecting evolutionary pressure to constrain lifetime mutational burden below levels that compromise organismal function (Cagan et al., 2022). Indeed, mutation accumulation can disrupt gene-regulatory networks, progressively impairing cellular functions and contributing to physiological decline as well as potentially to the fact that aging phenotypes are shared across individuals and species (Vijg and Suh, 2013).

#### 2.1.4 DNA repair mechanisms and age-dependent decline

Apart from the evidence of DNA damage accumulation with aging, the link between aging and DNA repair efficiency is further supported by the fact that several defects in DNA repair genes are associated with progeroid syndromes. In fact, the vast majority of molecular mechanisms causing progeroid syndromes are related to genome maintenance (Lombard et al., 2005) - deficiencies in DNA repair mechanisms have been demonstrated to accelerate aging in mice and contribute to human progeroid syndromes including Werner, Bloom, xeroderma pigmentosum, and others (López-Otín et al., 2013). Moreover, transgenic mice overexpressing BubR1, a mitotic checkpoint protein that ensures accurate segregation of chromosomes, demonstrate reduced aneuploidy, lower cancer risk, and extended healthy lifespan (López-Otín et al., 2013).

Genomic instability developing with aging is accompanied by downregulation of genes involved in DNA replication and repair, cell cycle progression, and control of mitosis (Ly et al., 2000), and it has been demonstrated that the efficiency of DNA repair mechanisms decreases with aging (Vijg and Suh, 2013; Freitas and De Magalhães, 2011). In *Caenorhabditis briggsae*, recent whole-genome transcriptome analysis revealed that DNA repair genes were upregulated during early reproduction (days 1–3), mildly declined by day 6, and significantly decreased in post-reproductive period (van den Berg and Gupta, 2025), suggesting that genomic instability plays a major role in aging. Decrease in DNA repair capacity when cells reach postmitotic state appears to be conserved across multiple species and has been demonstrated in mammalian muscle and neuronal cells (Gensler and Bernstein, 1981). Additionally, research on DNA polymerase α fidelity in cultured human fibroblasts indicates a nearly exponential rise in nucleotide misincorporation as cellular lifespan progresses (Kirkwood, 1989). These changes are an example of a process reconciling damage accumulation theory and evolutionary theories of aging, such as antagonistic pleiotropy; in this case–decrease of DNA repair mechanisms in postdevelopmental state may be beneficial early in life preserving resources for other cellular functions but become deleterious later in life due to the accumulated DNA damage (Gensler and Bernstein, 1981).

In particular, efficiency of double-strand break repair declines with age in budding yeast (McMurray and Gottschling, 2003) and mammals (Sedelnikova et al., 2004), as well as in senescent mammalian cells (Seluanov et al., 2004). Data mining analyses identified non-homologous end joining (NHEJ) as a key DNA repair pathway associated with aging (Freitas et al., 2011). And, as has been demonstrated in human cells, with aging, the mechanisms of double-strand break repair shift from simpler NHEJ processes to homologous recombination, and NHEJ becomes less accurate (Engels et al., 2007). It has been proposed that higher rate of employment of NHEJ in young organisms may represent an example of antagonistic pleiotropy allowing more rapid development, and thereby conducing a competitive advantage for most species, however, the use of error-prone pathways early in life may cause faster accumulation of DNA damage with negative consequences later in life (Engels et al., 2007). Several proteins involved in NHEJ, including KU70 and MRE11, exhibit age-related decline in expression in human hematopoietic stem and progenitor cells (Prall et al., 2007). These proteins also demonstrated statistically significant age-dependent changes in human lymphocytes, and KU70 was also found at higher levels in a long-lived South Korean population, and together with MRE11, was proposed as a potential biomarker of aging (Ju et al., 2006).

Gene expression analysis across 34 mammalian species revealed that homologous recombination gene expression correlates more strongly with longevity, while NHEJ gene expression correlates with encephalization, suggesting that enhanced NHEJ capacity may have coevolved with brain expansion to support double-strand break repair in postmitotic neurons (Udroiu and Sgura, 2024). NHEJ activity declines and double-strand breaks increase with age in rat cortical neurons (Freitas and De Magalhães, 2011). Additionally, NHEJ pathway defects impair hematopoietic stem cell function in older mice under stress conditions (Freitas and De Magalhães, 2011). The DNA damage response plays an important role in determining stem cell fate and potentially in exhausting the stem cell pool, being one of major triggers that drive cells into senescence, apoptosis and differentiation (Wolf, 2021a; Roos and Kaina, 2013; Hernandez-Segura et al., 2018; Inomata et al., 2009). p53-driven senescence and apoptosis in response to DNA damage play crucial role in the aging process (Lombard et al., 2005). *Loxodonta africana* have evolved an expanded repertoire of TP53 isoforms with diverse MDM2-binding motifs, enhancing cellular stress sensitivity and contributing to cancer resistance and longevity, highlighting adaptive coevolution of the p53–MDM2 axis as a key mechanism in large, long-lived mammals (Padariya et al., 2022). Another long-living mammal, the bowhead whale, which lives over 200 years with low cancer incidence, displays exceptionally efficient and accurate repair of DNA double-strand breaks and mismatches, and, consequently, lower mutation rate than other mammals (Firsanov et al., 2025). This phenotype is driven by high expression of CIRBP and its downstream effector RPA2, highlighting the importance of preserving genomic integrity for extreme mammalian longevity (Firsanov et al., 2025). Another example from comparative biology is the naked mole-rat, which displays enhanced homologous recombination repair linked to a unique cGAS variant (Chen et al., 2025). Four amino acid substitutions weaken TRIM41-mediated ubiquitination and P97 interaction, prolonging cGAS chromatin retention after DNA damage. This promotes FANCI-RAD50 interactions and RAD50 recruitment, supporting genome stability (Chen et al., 2025). These changes delay cellular and tissue aging, implicating DNA repair optimization in lifespan extension.

While cells from older adults generally demonstrate reduced repair capacity, cells from centenarians repair hydrogen peroxide-induced DNA strand breaks as effectively as those from young individuals (Chevanne et al., 2007). Consistent with this finding, PARP1 (the first responder to DNA damage) expression is significantly lower in older individuals but remains at levels comparable to the young in centenarians (Chevanne et al., 2007). Additionally, cells from centenarians exhibit low DNA damage, preserved telomeres, and reduced inflammation, partly via elevated RNAseH2C activity, which restrains DNA damage-induced interferon signaling and may underlie their resistance to chronic age-related inflammation (Storci et al., 2019).

#### 2.1.5 Mitochondrial DNA damage

It has been proposed that mitochondrial ROS production can damage mitochondrial DNA (mtDNA), which in turn can cause increased oxidant production by mitochondria, leading to a ‘vicious cycle’ (Balaban et al., 2005). This hypothesis was corroborated by a recent manuscript that demonstrated that oxidative damage contributes substantially to mtDNA mutations, as revealed by deconvoluted mutational signatures across chordates (Iliushchenko et al., 2025). Consistent with this finding, mitochondrial free radical generation has been demonstrated to be lower in long-lived compared to the short-lived species (Barja and Herrero, 2000). Mitochondria ROS production may cause damage not only to mtDNA itself, but to the nuclear DNA, which encodes most of the mitochondrial proteins. Therefore, when damage to the nuclear DNA is significant enough to cause genomic instability, it may create another ‘vicious cycle’ – when damaged nuclear DNA leads to impaired mitochondrial function, which in turn furthers nuclear DNA damage (Balaban et al., 2005).

While damage to the mitochondrial genome appears to be less consequential than damage to the nuclear DNA since mtDNA is present in thousands of copies (Lombard et al., 2005), the damage to mtDNA contributes to nuclear genomic instability (Barja, 2019). With aging fragments of mtDNA accumulate within nuclear DNA mainly at the pericentromeric regions through reverse transcription mediated by retrotransposons (Barja, 2019). These fragments can cause aneuploidy due to chromosome mis-segregation, genomic instability, cancer, and have been demonstrated to promote aging in model organisms (Barja, 2019).

The presence of multiple mtDNA copies per cell, however, does not exclude possibility of mtDNA mutations *per se* being an additional primary driver of aging. Unlike nuclear DNA, mtDNA lacks protective histones and is in close proximity to ROS-generating sites within the inner mitochondrial membrane, making it particularly vulnerable to damage (Whitehall et al., 2023). Experimental models, such as the PolgA mutator mouse harboring a proofreading-deficient mitochondrial DNA polymerase γ, recapitulate aging-like phenotypes including sarcopenia, anemia, and reduced lifespan, supporting a causative role (Whitehall et al., 2023). mtDNA mutations accumulate with age and have been implicated as a primary contributor to age-related dysfunction since pathogenic mutations can clonally expand and dominate within individual cells or even entire cell lineages (Whitehall et al., 2023). This expansion undermines the assumption that mitochondrial heteroplasmy dilutes the functional consequences of mtDNA damage. In fact, clonal expansion allows a single mtDNA mutation to reach high local abundance, leading to respiratory chain deficiency in specific tissues (Whitehall et al., 2023). For example, in aging human skeletal muscle, cytochrome c oxidase (COX)-deficient fibers increase with age due to the accumulation of clonally expanded mtDNA deletions (Vijg et al., 2015). These fibers often contain a homoplasmic or near-homoplasmic population of mutated mtDNA, leading to focal regions of mitochondrial dysfunction that compromise contractile strength and regenerative potential (Whitehall et al., 2023). A similar phenomenon occurs in the colonic epithelium, where mtDNA deletions can clonally populate entire colonic crypts (Vijg et al., 2015). Each crypt originates from a small number of stem cells, and once a mutation arises and gains dominance in a stem cell, its progeny can fill the entire crypt over time (Vijg et al., 2015). These COX-deficient crypts increase in number with age and may impair epithelial turnover and barrier integrity. The existence of such clonally expanded mitochondrial defects in post-mitotic and proliferative tissues indicates that mtDNA damage is not only prevalent but also subject to selective dynamics that exacerbate its impact (Whitehall et al., 2023). These expansions occur despite ongoing mitochondrial biogenesis and turnover, pointing to inadequate quality control or biased replication favoring mutated genomes (Whitehall et al., 2023). The tissue-specific accumulation of mtDNA mutations through clonal expansion may therefore represent a critical mechanism linking mtDNA mutations with mitochondrial dysfunction and progressive decline in organ function observed during aging.

#### 2.1.6 Telomeric DNA damage

Telomeric DNA damage is also contributing to the aging process, and telomeric DNA repair efficiency decreases with age (Kruk et al., 1995; Goyn and s, 2002). In multiple species including humans, telomeric DNA is especially vulnerable to damage due to its high susceptibility to oxidative damage and interference of protective telomere-binding proteins with repair; that results in persistent damage signaling that requires telomerase to resolve (Blackburn et al., 2015). Inducing oxidative 8-oxo-guanine lesions at telomeres in human cells triggered p53-dependent senescence via replication-dependent telomere fragility and increased cytoplasmic DNA, a known driver of inflammation, without telomere shortening or shelterin loss, demonstrating that oxidative base damage alone without attrition can rapidly induce telomere dysfunction and cellular senescence (Barnes et al., 2022).

Telomerase is crucial for telomere maintenance not only by preventing critical telomere shortening but also by reducing DNA damage associated with the loss of telomere integrity. Studies have demonstrated that in cells cultured without telomerase, the frequency of telomere fusions to DNA breaks is significantly higher than in wild-type controls. Notably, these fusions remain elevated even when telomeres were pre-elongated before telomerase deletion, highlighting its role beyond telomere extension (Chan and Blackburn, 2004).

The role of telomeres in aging biology goes beyond telomere attrition and damage causing cellular senescence - it has been discovered that telomeric non-coding RNAs (TERRA) and telomeric damage-induced long noncoding RNAs play roles in the ageing process as both elevated and decreased TERRA levels may impair telomere homeostasis (Aguado et al., 2020). Additionally, TERRA were found to accumulate in premature ageing conditions and trigger DNA damage responses at telomere sites (Aguado et al., 2020).

Together, damage to nuclear (including telomeric) and mitochondrial DNA establishes genomic instability as a core upstream driver of aging, facilitating mutation accumulation, transcriptional dysfunction, stem-cell exhaustion, and senescence that propagate aging through reinforcing feedback loops.

### 2.2 Telomere attrition

Telomere attrition is a hallmark of aging which causes replicative senescence via persistent DNA damage response (Kruk et al., 1995). Telomeres preserve chromosome integrity but progressively shorten with each replication in the absence of telomerase. Telomere dysfunction and its consequences, including cell cycle arrest and apoptosis, are driven not by average telomere length, but by critically short telomeres, which trigger chromosomal instability and limit cell survival in telomerase-deficient mouse models (Hemann et al., 2001). Telomere shortening also induces long-range transcriptional changes through the telomere position effect over long distances, altering expression of distal genes prior to critically short telomere formation (Robin et al., 2014). Although telomerase restores telomeric repeats, its expression in humans is largely restricted to embryonic development and particular adult stem cell populations, resulting in gradual telomere erosion in most somatic cells. In most human somatic cells, telomerase is downregulated after differentiation through mechanisms such as TERT transcriptional silencing (Chiba et al., 2015) and alternative splicing (Penev et al., 2021), unlike in embryonic and induced pluripotent stem cells where it maintains telomere length for continuous proliferation. Therefore, due to repressed telomerase activity, most human somatic cells undergo gradual telomere shortening (Lansdorp, 2022). Critically short telomeres lose their protective cap, triggering DNA damage response that enforces replicative senescence (Risques and Promislow, 2018), a mechanism thought to have evolved as a cancer-suppressive strategy (Campisi, 2001). However, loss of telomerase activity is associated with aging in both humans and model organisms (Razgonova et al., 2020; Rossiello et al., 2022), and ectopic expression of telomerase in human cells *in vitro* has been demonstrated to prevent replicative senescence (Chan and Blackburn, 2004). Cells with defective DNA damage responses, such as p53 loss, can bypass proliferation limits imposed by telomere erosion, accelerating tumor progression through genomic instability (Lansdorp, 2022).

In most species, telomerase expression and telomere length are influenced by body size. Short telomeres are favored for tumor suppression, while long telomeres help counteract DNA damage and replicative senescence. The balance between these selective pressures depends on species-specific cancer risk and intrinsic mortality, both of which correlate with body size (Risques and Promislow, 2018). In mammals, telomerase is silenced after embryonic development in species over ∼2 kg, while smaller species often retain its expression. Animals above ∼5 kg typically have short telomeres, while smaller species demonstrate higher variation. It has been hypothesized that common mammalian ancestor likely repressed telomerase expression in adults as an adaptation to homeothermy and the resulting increase in mutation burden, with later independent reactivations during evolutionary process (Gomes et al., 2011). Inbred mouse strains are highly prone to cancer (the main mortality cause in these mice) and have telomeres up to ten times longer than humans, consistent with the theory that in humans telomere shortening had developed as a mechanism for preventing cancer via limiting excessive cell divisions (Wright and Shay, 2000). Telomere shortening in human cells is accelerated by increased oxidative stress (Lombard et al., 2005), yet species with long telomeres often exhibit lower oxidative-protection mechanisms, which may represent an evolutionary trade-off that favors constitutive telomerase activity and reduced dependence on energetic antioxidant defenses (Gomes et al., 2011).

Telomere attrition is associated with human morbidity and mortality (Von Figura et al., 2009). Greater telomere attrition predicts increased mortality and higher risk of aging-related diseases both in individuals with inherited telomere syndromes and in the general population (Blackburn et al., 2015). Human mortality rates from diverse causes, including cardiovascular disease and infections, are higher in elderly individuals with shorter leukocyte telomere length compared to those with longer telomeres (Chan and Blackburn, 2004). Telomere attrition contributes to age-associated decline in stem cell function, and age-related telomere shortening has been detected in human CD34^+^ hematopoietic stem and progenitor cells (Von Figura et al., 2009). While telomere attrition in humans presumably has evolved as cancer-protective mechanism, individuals born with shorter-than-average telomeres face elevated cancer risk, a phenomenon known as the “telomere paradox”, potentially due to selective expansion of abnormal stem cells and eventual failure of telomere-based tumor suppression (Nassour et al., 2021).

The most common disorders associated with telomere attrition are bone marrow failure in young patients and pulmonary fibrosis due to telomerase haploinsufficiency in individuals after puberty (Lansdorp, 2022). Inherited mutations in telomerase components (*TERT, TERC, DKC1, NHP2, NOP10, WRAP53*) or telomere-binding proteins, which were proven to have essential telomere-protecting functions (*CTC1, POT1, TINF2, RTEL1, TPP1*), compromise telomere maintenance, leading to progressive telomere shortening and loss of chromosomal end protection (Blackburn et al., 2015). These defects manifest as inherited telomere syndromes, characterized by pulmonary fibrosis, hematologic and epithelial cancers, liver cirrhosis, gastrointestinal disorders, immune dysfunction due to loss of hematopoietic stem cells, neuropsychiatric disorders, and premature aging phenotypes (Blackburn et al., 2015). Disease severity increases across generations due to genetic anticipation, and clinical symptoms can appear even in non-mutant offspring inheriting critically short telomeres (Blackburn et al., 2015). These inherited telomere syndromes present early and severely but manifest as common age-related diseases such as immune decline, cancer, diabetes, and cardiovascular disease - the same conditions associated with shorter telomeres in leukocytes and peripheral blood mononuclear cells, and driven, in part, by pro-inflammatory processes arising from immune cell senescence due to telomere attrition (Blackburn et al., 2015). For example, telomere length has been implicated in common age-related diseases such as cardiovascular and Alzheimer’s disease, with genetic studies linking shorter telomeres to increased risk for both conditions (Blackburn et al., 2015).

While inherited upregulation of telomerase has been implicated in increased risk of some cancers, environmental influences preventing telomere attrition, such as lower psychological stress, non-smoking, and exercise, have been demonstrated to render the opposite effect on cancer development (Blackburn et al., 2015).

Experimental model systems provide additional evidence that telomere erosion is a primary driver of aging. In telomerase-knockout mice, activation of DNA damage checkpoints contributes to the decline in stem cell populations (Von Figura et al., 2009). Constitutive telomerase expression, combined with overexpression of tumor suppressors p53, p16, and p19ARF, extends median lifespan and delays aging in cancer-resistant mice (Tomás-Loba et al., 2008). This strategy improves epithelial maintenance, particularly in the skin and intestine, supporting a role for telomerase in promoting longevity under conditions of cancer suppression (Tomás-Loba et al., 2008). In laboratory mouse models, telomerase deficiency alone results in pathological phenotypes only after several generations due to long baseline telomeres. However, in compound models combining telomerase deletion with other mutations, such as *Wrn* (Werner syndrome), *Blm* (Bloom syndrome), *Ins2C96Y* (Akita diabetes), or dystrophin (Duchenne muscular dystrophy), disease phenotypes emerge earlier and are more severe (Blackburn et al., 2015). These models demonstrate synergistic interactions between telomere dysfunction and defects in genome maintenance, ER stress, and structural protein loss, revealing telomere erosion as a sensitizing factor for multiple diseases, in particular, diseases associated with defects in DNA repair (Blackburn et al., 2015).

Comparative biology data suggests relevance of telomere dynamics to the aging process. Across 19 bird species, long-lived birds like ostriches and flamingos demonstrated slower telomere shortening rates, suggesting that telomere maintenance co-evolved with species-specific longevity (Tricola et al., 2018), and in Myotis bats, telomeres are maintained with age despite potential lack of telomerase expression due to alternative telomere maintenance and enhanced DNA repair (e.g., ATM, SETX) as evolutionary adaptations for longevity (Foley et al., 2018).

Although tightly interconnected with other pathways, telomere attrition appears to function as a primary aging mechanism because critically short telomeres directly trigger DNA damage responses, senescence, chromosomal instability, and long-range transcriptional disruption. These effects arise independently of, yet reinforce, DNA damage itself, creating redundant and self-amplifying drivers of aging. In humans, where telomerase is largely repressed and lifespan is long, progressive telomere shortening becomes a predictable upstream constraint on stem-cell function and tissue maintenance, positioning telomere erosion as one of the core primary drivers of aging.

### 2.3 Sex hormone decline

Hormonal decline is another change occurring with aging (Hertoghe, 2005); however, whether sex hormone decline represents a primary aging mechanism or a downstream consequence of molecular damage likely depends on biological context. In humans, female reproductive aging driven by progressive depletion of ovarian reserve represents a biologically programmed process that occurs independently of somatic molecular damage accumulation and may function as a sex-specific primary driver of aging-related physiological decline (Blagosklonny, 2010; Xing Y. et al., 2023; Jaschke et al., 2021; Finch, 1994). Additionally, while testosterone depletion has been demonstrated to extend life in males across multiple vertebrate species (Garratt et al., 2025) including humans (Min et al., 2012), estrogen is geroprotective (Borrás et al., 2021), as it influences all 12 hallmarks of aging (R et al., 2025), therefore this section will focus on the evidence of female sex hormone decline as a primary driver of aging. It is important to note, however, that because menopause is specific to females and varies across species, classification of sex-hormone decline as a primary mechanism applies specifically to female aging in humans.

A higher germline mutation rate in young adults is linked to increased mortality and, in women, to reduced fertility and earlier reproductive decline (Cawthon et al., 2020). While this rate may serve as an early indicator of systemic and reproductive aging, likely becoming established at puberty - when DNA repair capacity begins its lifelong deterioration (Cawthon et al., 2020), limited ovarian reserve in females elicits reproductive aging independent of DNA damage and telomere attrition.

Hormonal decline is not currently listed as a hallmark of aging and in available literature is rarely described as a primary aging mechanism, yet, in females, menopause independently drives aging and accelerates many aging-related diseases (de Magalhães, 2025b). While most mammals do not undergo abrupt menopause which observed in humans, decreased ovarian reserve occurring with aging and resulting decline of sex hormone levels is shared by most mammals (Finch, 1994), including mice (Hense et al., 2024), in which estrogen levels have been demonstrated to significantly decrease with age, and most female mice become acyclic by the age of 25 months (Frick, 2008).

Hormonal decline is a key feature of aging, and ovarian reserve depletion leads to systemic metabolic and physiological changes that contribute to accelerated aging and increased disease risk (Finch, 1994; Hense et al., 2024; Nair and Jacob, 2016; Moran et al., 2007; Baik et al., 2024; Lundberg et al., 2020; Tartagni et al., 2007). Menopause markedly increases the risk of death and accelerates the onset of major diseases such as cardiovascular disease, diabetes, and osteoporosis (Ossewaarde et al., 2005; Zhu et al., 2019; Huang et al., 2023; Xing Z. et al., 2023; Asllanaj et al., 2019; Hense et al., 2024). Furthermore, raising before menopause, elevated follicle-stimulating hormone (FSH) levels, independently from estrogen levels, are associated with multiple adverse health outcomes including increased hip fracture risk (Koh et al., 2024), hypercholesterolemia (Guo et al., 2019), higher BMI and lower lean mass (Veldhuis-Vlug et al., 2021), as well as increased biomarker risk of Alzheimer’s disease (Nerattini et al., 2023).

Human aging exhibits pronounced sexual dimorphism, with proteomic aging clocks identifying distinct age-related trajectories and protein signatures in males and females. In a recent large-scale proteomic analysis (Jin et al., 2025), females demonstrated a more nonlinear aging trajectory than males, with a marked inflection point aligning with the menopausal transition. This divergence may reflect the abrupt endocrine collapse unique to human female aging. In contrast, male proteomic aging appeared more linear and was associated with a greater number of mortality-linked proteins (1 in females vs. 172 in males) (Jin et al., 2025). These findings support the hypothesis that hormonal decline, particularly estrogen loss, plays a central and sex-specific role in shaping aging trajectories and biological risk profiles.

Multiple mechanistic evidence favors considering female sex hormone decline as one of the primary drivers of aging:

- The fact that estrogen stimulates a promoter of the telomerase was proposed as one of the explanations why mammalian females live longer than males (Kyo et al., 1999). It could also potentially explain that most published studies report that adult females tend to have longer telomeres than males (Fischer and Riddle, 2018), as well as the smaller decrease in lifespan when females are the heterogametic sex - homogametic sex lives 7.1% longer in female heterogametic species but 20.9% longer in male heterogametic species (Xirocostas et al., 2020).
- Estrogen-related receptor alpha (ERRα) is a central regulator of mitochondrial biogenesis and oxidative metabolism (Ranhotra, 2015), and its transcriptional activity is strongly dependent on the coactivator PGC-1α (Au et al., 2016). Estrogen signaling through ERα upregulates PGC-1α expression and activity, thereby potentiating ERRα-mediated mitochondrial gene expression. With menopause, loss of estradiol signaling decreases ERα-driven PGC-1α activation, leading to reduced ERRα-dependent mitochondrial transcriptional programs (Klinge, 2020). This provides a mechanistic route through which menopausal endocrine collapse contributes to mitochondrial dysfunction.
− 17β-estradiol (E2) activates NRF2/ARE programs and increases antioxidant enzyme expression and Nrf2 nuclear translocation (Yu et al., 2012). Consistent with this, estrogen deficiency causes oxidative stress and DNA damage in rat models (Tang et al., 2012; Wassmann et al., 2001).
- A large metagenomic study of over 2,300 Hispanic/Latino adults found that postmenopausal women exhibit a gut-microbiome profile with reduced diversity and taxa shifts that resemble those of men, alongside functional changes in microbial hormone-processing pathways (Peters et al., 2022). These menopause-associated microbiome alterations correlate with worse cardiometabolic markers, suggesting that loss of ovarian hormones reshapes gut ecology in ways linked to metabolic risk (Peters et al., 2022).
- Additionally, estrogen deficiency during menopause disrupts multiple aging-related processes through coordinated failures in central and peripheral homeostasis. Loss of ERα signaling in key hypothalamic nuclei impairs regulation of food intake, thermogenesis, and energy expenditure, producing weight gain, altered metabolic rate, and reduced physical activity (Camon et al., 2024). Peripheral estrogen loss further destabilizes metabolic homeostasis: hepatic ERα decline alters IGF-1 and glucose regulation and increases susceptibility to steatosis, while adipose ERα loss promotes visceral adiposity, inflammation, and fibrosis (Camon et al., 2024). Declining estrogen signaling in bone shifts remodeling toward osteoclast-driven resorption, accelerating osteoporosis, and in skeletal muscle reduces insulin sensitivity and mitochondrial function, impairing strength and repair (Camon et al., 2024).

Therefore, estrogen depletion propagates secondary hallmarks and manifestations of aging including altered intercellular communication, immune dysregulation, metabolic inflexibility, and impaired tissue maintenance, supporting the interpretation that ovarian endocrine decline may function as a major sex-specific upstream driver of aging-related physiological deterioration in humans.

### 2.4 Damage to proteins and lipids

While less impactful than damage to the DNA (since other molecules are present in multiple copies in the cells and most are recycled regularly), mechanisms acting as both primary and secondary include damage to molecules other than DNA including protein damage with loss of proteostasis and lipid damage, which may arise directly from molecular insults or emerge as downstream consequences of DNA damage, telomere attrition, and hormonal changes, while also feeding back to accelerate upstream deterioration.

#### 2.4.1 Protein damage and loss of proteostasis

Protein damage and dysregulated proteostasis represent both primary and secondary mechanism of aging. As a primary mechanism, it arises from cumulative damage to intrinsically unstable aggregation-prone and long-lived proteins due to oxidative stress, glycation, and spontaneous chemical modifications (Paull, 2021; Hipp et al., 2019). Long-lived proteins accumulate irreversible structural defects, while aggregation-prone proteins misfold and form toxic assemblies when chaperone capacity is exceeded or clearance pathways fail (Paull, 2021). Protein misfolding and aggregation can exacerbate genomic instability by elevating oxidative stress, impairing mitochondrial function, disrupting chromatin structure, and impairing DNA maintenance through sequestration of repair factors (Xie and Jarosz, 2018). For example, protein aggregates can interfere with DNA damage responses by sequestering or inactivating essential DNA repair proteins such as p53, thereby compromising genome maintenance (Xie and Jarosz, 2018).

A proof of principle example demonstrating partial primary role of protein damage in the aging process comes from *E. coli* model, where protein damage compromised cellular function, leading to elevated mutation rates and progressive functional decline resembling aging - protein carbonylation promoted a mutator phenotype, while reducing it below wild-type levels created an anti-mutator effect, indicating its role in spontaneous mutations (Krisko and Radman, 2013). Additionally, protein oxidation increased UV-induced mutagenesis and disrupted biosynthesis, impairing cellular processes (Krisko and Radman, 2013). Using *E. coli* strains with altered protein quality control systems, it has been demonstrated that these effects occur independently of direct DNA damage, as oxidative DNA lesions remained constant. Antioxidant administration reduced protein oxidation, mitigating its mutagenic consequences and preserving cellular function (Krisko and Radman, 2013). Although bacterial proteostasis differs from eukaryotic systems, the causal link between protein oxidation and elevated mutation rates supports a primary role in destabilizing genomic integrity.

Concurrently, proteostasis failure is a secondary consequence of genomic and epigenomic instability, as DNA mutations, transcriptional errors, and chromatin alterations disrupt the synthesis, folding, and degradation of proteins by impairing molecular chaperones, autophagy, and the proteasomal system (Paull, 2021; Meller and Shalgi, 2021). DNA damage and mutations can compromise proteostasis by reducing transcriptional and translational fidelity, altering protein coding sequences, and producing structurally abnormal or aggregation-prone proteins that are more likely to misfold, escape proper folding, and overwhelm the proteostasis network (Paull, 2021; Xie and Jarosz, 2018). Therefore, mutagenesis resulting from genomic instability leads to transcriptional noise and production of misfolded proteins, contributing to proteome instability and proteotoxic stress (Xie and Jarosz, 2018). DNA damage responses involving p53, ATM, and related signaling pathways regulate the expression of proteostasis components such as chaperones and autophagy regulators, linking genome instability to proteome collapse (Paull, 2021). Additionally, in osteoblasts, estrogen directly supports proteostasis by promoting cytoplasmic localization of the epigenetic regulator Setdb1, which stabilizes the collagen-folding chaperone Serpinh1; estrogen loss in ovariectomized mice provokes unfolded-protein accumulation and ER stress, demonstrating a mechanistic link between estrogen deficiency and impaired protein homeostasis (Han et al., 2025).

Taken together, these reciprocal interactions justify classifying proteome deterioration as both primary and secondary aging mechanism.

#### 2.4.2 Lipid damage

Similar to the protein damage, lipid damage can represent both primary and secondary aging mechanism. At the primary level, membrane polyunsaturation enables chain-reaction peroxidation that generates reactive carbonyl species capable of forming stable adducts and crosslinks with proteins. These modifications accumulate as lipofuscin and other long-lived lipoxidation end-products, which progressively impair cellular homeostasis (Jové et al., 2020). Additionally, products of lipid peroxidation such as reactive aldehydes (hydroxynonenal and malondialdehyde) can diffuse through membranes and form adducts with DNA, contributing to nuclear and mitochondrial DNA damage. These aldehydes may also cause DNA-protein crosslinks, potentially hindering gene expression (Barja, 2019).

At the secondary level, genomic instability and declining stress-response capacity weaken antioxidant defenses, increasing peroxidation susceptibility and accelerating molecular injury. Low levels of membrane fatty acid unsaturation in long-lived species reduce susceptibility to lipid peroxidation, highlighting the evolutionary importance of intrinsic membrane composition in limiting oxidative macromolecular damage during aging (Pamplona et al., 2002). Accordingly, comparative and interventional evidence suggests that low-unsaturation membranes correlate with extended longevity, while experimental increases in membrane unsaturation elevate lipid peroxidation and reduce lifespan in models such as mice and *C. elegans* (Jové et al., 2020). These findings indicate that lipid damage functions as both a primary generator of reactive molecular injury and a secondary amplifier of damage arising when protective and repair systems decline.

## 3 Secondary and tertiary mechanisms

### 3.1 Secondary mechanisms

Secondary aging mechanisms can be traced back to core upstream pressures, such as damage to DNA and other molecules, telomere attrition, and hormonal changes. It has been hypothesized that some alterations representing hallmarks of aging directly ensue from primary mechanisms, while the others are the response/adaptation to these primary mechanisms (Hayflick, 2007; López-Otín et al., 2013; López-Otín et al., 2023; Aunan et al., 2016; Hambright et al., 2019; Rattan, 2008; Holliday, 2006). While factors outlined below can further exacerbate the initial triggers, their impact tends to be downstream or context-dependent rather than representing initiating drivers of aging.

Although **epigenetic changes** accumulate with age (Soto-Palma et al., 2022) and serve as powerful biomarkers of aging (Horvath and Raj, 2018), their progressive accumulation is strongly influenced by upstream factors such as DNA damage (Soto-Palma et al., 2022). The adaptive nature of these modifications is underscored by their functional roles in the DNA damage response; for example, acetylation/deacetylation dynamics determine choice of the DNA double-strand break repair pathway (Soto-Palma et al., 2022), and DNA damage has been demonstrated to induce epigenome instability (Soto-Palma et al., 2022; Russo et al., 2016). Additionally, recent evidence indicates that age-associated DNA methylation drift originates within tissue stem cells and is driven by inflammatory signaling and altered niche cues rather than replication-dependent processes, supporting epigenetic change as a downstream response to upstream aging pressures rather than an initiating mechanism (Krepelova et al., 2025). Thus, while epigenetic alterations can arise both as direct consequences of DNA damage and as adaptive mechanisms supporting repair (Ying et al., 2024), epigenetic drift is primarily downstream of DNA damage, but once established, these alterations can further impair expression of DNA repair genes, antioxidant systems, and chromatin integrity, creating a feedback loop that exacerbates genomic instability (Xie and Jarosz, 2018). For example, loss of heterochromatin and histones in aging yeast cells promotes transcriptional dysregulation and contributes to the development of genomic instability, increasing DNA breaks, translocations, and mitochondrial DNA insertions (Pa et al., 2016). Long-lived mammals appear to have evolved both sequence-level and methylation-based strategies to suppress mutagenic DNA structures, suggesting that the erosion of these protective mechanisms with age contributes to genomic instability (Smith, 2025). Human DNA methylation studies further indicate that epigenetic alterations generally progress more slowly in females than in males across most tissues (Fischer and Riddle, 2018), suggesting sex-specific aging trajectories and hormonal profile influences. Although some epigenetic alterations are developmentally programmed (John and Rougeulle, 2018), and others may represent spontaneous epigenetic drift (Teschendorff et al., 2013), strong evidence for epigenetic changes as independent, primary aging drivers in the absence of DNA damage remains limited. Overall, most aging-associated epigenetic changes appear to be causally downstream of DNA damage and its cellular consequences (Soto-Palma et al., 2022; Siametis et al., 2021), acting more as mediators of aging rather than initiators, in contrast to the highly regulated epigenetic remodeling that characterizes early development.

**Cellular senescence** is the state of irreversible cell cycle arrest, when cells remain metabolically active and acquire senescence-associated secretory phenotype (SASP) characterized by secretion of inflammatory mediators, interleukins, cytokines, matrix metalloproteinases and growth factors, contributing to chronic inflammation observed in aging (Coppé et al., 2010). It has been proposed that cellular senescence acts as an adaptation to the molecular damage, in particular, DNA damage (Schumacher et al., 2008; Hayflick, 2007; Yousefzadeh et al., 2021; Schumacher et al., 2021; Kirkwood, 2008), as well as telomere attrition (Yousefzadeh et al., 2021; Schumacher et al., 2021) potentially decreasing cancer incidence early in life through preventing proliferation of damaged cells, but driving aging related degeneration later in life through SASP which can maintain proinflammatory milieu and cause paracrine senescence, leading to decrease in tissue proliferative potential (Nelson et al., 2012). While transient senescent cell upregulation plays beneficial roles during development and regeneration (Shvedova et al., 2022), chronic accumulation of senescent cells contributes to aging-associated diseases and even increases cancer risk later in life (Vijg, 2021; Vijg, 2014; Campisi, 2003a; Campisi, 2003b; Gems and Kern, 2022). Accordingly, a strong positive correlation was detected between the proportion of cells in a population that entered senescence after DNA damage and the species’ lifespan (Attaallah et al., 2020).

Mitochondria-derived oxidative stress together with genomic and mtDNA mutations drive progressive **mitochondrial dysfunction** with age, including increased proton leak, impaired cardiolipin-dependent protein function, and diminished ATP production, which together lead to widespread cellular deterioration and implicate mitochondrial damage as an important self-amplifying contributor to organismal aging (Shigenaga et al., 1994). Heterozygous inactivation of the mitochondrial gene mclk1, the mammalian ortholog of clk-1, extends lifespan in mice and confers resistance to oxidative stress and DNA damage, highlighting an evolutionarily conserved, insulin-independent mechanism of aging modulation via altered ubiquinone biosynthesis and acting through decrease in molecular damage (Liu et al., 2005).

Aging is associated with **altered intercellular communication**, including increased proinflammatory cytokine release, impaired endocrine responsiveness, and altered extracellular vesicle profiles. These changes reinforce senescence, inflammation, and mitochondrial dysfunction but arise downstream of primary drivers (López-Otín et al., 2023).

**Autophagy declines** with age through reduced autophagosome formation, impaired lysosomal degradation, and diminished cargo recognition. This loss of proteostatic clearance exacerbates protein aggregation and mitochondrial dysfunction and reflects a downstream consequence of persistent primary molecular drivers (López-Otín et al., 2013).

Nutrient-responsive pathways such as mTOR, AMPK, and insulin/IGF signaling lose adaptive responsiveness with age, altering metabolic flexibility and stress resistance (López-Otín et al., 2023). **Deregulated nutrient sensing** influence cellular fate decisions and proliferative behavior but generally represent secondary responses to accumulated genomic, metabolic, and hormonal stress rather than initiating drivers of aging.

The distinction between primary and secondary mechanisms is inherently fluid, since processes such as protein and lipid damage or epigenetic alterations can originate either as direct molecular insults or as downstream changes, and multiple feedback loops exist; however, the hierarchical framework allows for a mechanistic separation of the processes that initiate molecular damage from those that amplify, propagate, or manifest its downstream effects.

### 3.2 Tertiary mechanisms

Tertiary mechanisms represent late-stage functional declines that arise after accumulation of upstream molecular damage and secondary cellular responses, reflecting consequences rather than initiators of the aging process.

**Stem cell exhaustion** has been demonstrated to result from DNA damage (Schumacher et al., 2021), representing a key downstream consequence of age-related genomic instability (Sharpless and DePinho, 2007; De Magalhães and Faragher, 2008). During ageing, accumulated telomere dysfunction and genomic instability contribute to a decline in stem-cell self-renewal and differentiation capacity (Ermolaeva et al., 2018). Sex-hormone decline also contributes to stem-cell dysfunction as estrogen signaling through ERα directly promotes hematopoietic stem-cell division and regenerative output, an effect observed in both females and males (Nakada et al., 2014). Loss of estradiol signaling with menopause therefore removes an important proliferative cue supporting stem-cell maintenance.

Chronic, low-grade **inflammation (inflammaging)** is a pervasive feature of aging and emerges as a tertiary consequence of accumulated cellular damage, senescence, mitochondrial dysfunction, and impaired proteostasis. Age-related shifts in cytokine networks, persistent innate immune activation, and diminished resolution of inflammation contribute to a sustained proinflammatory state that accelerates tissue degeneration. Inflammaging is reinforced by multiple secondary processes, particularly the senescence-associated secretory phenotype, which amplifies oxidative stress, alters intercellular communication, and feeds back onto genomic and epigenetic instability (López-Otín et al., 2023; Coppé et al., 2010).

Aging is also accompanied by progressive alterations in gut microbial composition, with reduced diversity, loss of beneficial commensals, expansion of pathobionts, and impaired epithelial barrier integrity (Ling et al., 2022). These changes further promote systemic inflammation, disrupt nutrient and metabolite signaling, and contribute to immune dysregulation, but arise as a tertiary manifestation of upstream molecular damage that compromises host-microbe homeostasis (Ling et al., 2022). **Dysbiosis** interacts bidirectionally with chronic inflammation and metabolic dysfunction, further propagating oxidative stress and exacerbating tissue-specific aging phenotypes (López-Otín et al., 2023).

While some aging phenotypes may have tissue-specific thresholds or dynamics, they ultimately arise through shared upstream pressures such as molecular damage, telomere attrition, and hormonal changes. Skeletal muscle aging exemplifies how systemic damage culminates in tissue-specific phenotypes. Neuromuscular junction degeneration and anabolic resistance, for instance, are manifestations of mitochondrial dysfunction, impaired proteostasis, oxidative stress, and disrupted nutrient sensing in muscle and motor neurons (Gonzalez-Freire et al., 2014).

Importantly, multiple feedback loops exist in which secondary and tertiary mechanisms reinforce the primary drivers. For example, cellular senescence promotes chronic inflammation through SASP (Coppé et al., 2010); this inflammatory milieu induces endoplasmic reticulum stress and elevates reactive oxygen species, which in turn accelerate DNA damage and reinforce genomic instability and epigenetic changes (Pa et al., 2016).

## 4 Implications of redundancy of primary mechanisms for healthspan and lifespan extending interventions

Research aimed at extending healthspan and ultimately lifespan has been gaining traction during the recent years, and multiple pathways representing potential targets have been described to influence the aging phenotypes in model organisms, such as DNA damage response, telomere repair, Nuclear Factor Kappa B pathway, heat shock proteins, mitochondrial pathways, insulin signaling pathway, sirtuins, and others (Ljubuncic and Reznick, 2009). Caloric restriction is one of the most consistently studied interventions targeting aging pathways, yet its effects in mammals remain limited and context dependent. While caloric restriction produces modest lifespan extension in some mouse strains (Di Francesco et al., 2024), most positive findings derive from simpler organisms or progeroid murine models that do not reflect natural aging (Kõks et al., 2016; Harkema et al., 2016). Lifespan responses are strongly genotype dependent, with dietary restriction shortening lifespan in more strains than it extends it (Liao et al., 2010). Studies in primates have also yielded conflicting results, with some reporting health benefits but no clear lifespan extension (Mattison et al., 2012). These discrepancies may reflect the fact that caloric restriction often counteracts laboratory overfeeding rather than producing a direct anti-aging effect (Wolf, 2021a; Wolf, 2021b). Regardless, mechanistically, caloric restriction appears to reduce damage accumulation by decreasing reactive oxygen species, lowering oxidative DNA and protein damage, improving DNA repair (Barja, 2019; Heydari et al., 2007; Pamplona and Barja, 2006; Garcia et al., 2008), and reducing proliferation rate (Wolf, 2021a) hence attenuating aging-associated telomere erosion (Vera et al., 2013). It also reduces mitochondrial ROS and mtDNA damage through mechanisms observed in long-lived species (Barja, 2002) and enhances autophagy (Barja, 2019), which itself can extend lifespan in murine models (Pyo et al., 2013). Overall, although caloric restriction modulates several upstream aging-related pathways, its limited and inconsistent effects on mammalian lifespan are consistent with the presence of multiple, parallel primary drivers of aging that cannot be durably constrained by a single intervention, particularly when initiated after substantial damage has already accumulated.

Considering the multifaceted nature of aging, any single-target interventions are unlikely to produce meaningful healthspan and lifespan extension (Beard et al., 2016). Yet complex preventive strategies have been underexplored on the translational aging research landscape. To date no intervention targeting aging has been conclusively proven to extend human lifespan (Pitt and Kaeberlein, 2015). Approaches using combined treatments aimed at healthspan extension remain rare even in preclinical settings and to date they have focused on rejuvenation rather than preventing damage accumulation (Fahy et al., 2019; Lewis and de Grey, 2024; de, 2023; Kato et al., 2025). While the development of interventions aimed at repair is essential, such interventions may work best in combination with preventive strategies, as, for example, stem cell replacement would be more efficient in lower damage settings improving homing; and no approach has yet conclusively reversed the core upstream driver of aging–DNA damage (Schumacher et al., 2021; Sinclair and Oberdoerffer, 2009) – it can only be slowed down using either antioxidants (Varesi et al., 2022) or substances improving DNA repair precision (Bujarrabal-Dueso et al., 2023). Therefore, combining preventive strategies with rejuvenation may achieve the most robust healthspan and lifespan extension (Lewis and de Grey, 2024).

The timing of preventive interventions represents an important consideration as while clinical manifestations of aging typically emerge later in life, accumulating evidence indicates that aging-associated molecular alterations originate much earlier, including during embryonic development and postnatal growth, when epigenetic and molecular trajectories associated with aging are established (Gladyshev, 2020; Kerepesi and Gladyshev, 2023). In this context, aging is considered a lifelong process beginning with the establishment of molecular states that influence long-term maintenance and repair capacity. Accordingly, preventive strategies may be most beneficial if initiated prior to substantial accumulation of irreversible molecular damage, generally encompassing early adulthood before overt functional decline becomes clinically apparent. The following part of this section provides the rationale and potential modalities for early, multi-component preventive strategies targeting redundant primary aging mechanisms and reviews recent complex rejuvenation interventions and their limitations. Table 1 provides a summary of the potential interventions discussed. Long-term studies evaluating early preventive interventions targeting individual mechanisms as well as combined approaches will be necessary to determine the feasibility, safety, and efficacy of the proposed preventive strategy.

**TABLE 1:** Interventions targeting hypothesized primary aging mechanisms.

Target	Intervention	Proposed mechanisms and published outcomes
Damage accumulation	Exogenous lipophilic antioxidants (e.g., astaxanthin)	Improves mitochondrial function (Yu et al., 2019; Sun et al., 2021; Sztretye et al., 2019) Non-pro-oxidant profile (Maezawa et al., 2016) Increases expression of endogenous antioxidant enzymes (Wang et al., 2022; Monmeesil et al., 2019) Decreased oxidative DNA damage (Park et al., 2010) and reduced skin aging in humans (Belviranl et al., 2015) Lifespan extension in male mice in ITP (Harrison et al., 2024)
	Mitochondria-targeting antioxidants (e.g., MitoQ, SkQ1, MitoTEMPO, and XJB-5-131)	Selective accumulation in mitochondria Reduction of mitochondrial ROS Decreased DNA damage and cellular senescence markers in preclinical models (Abdeahad et al., 2025)
	Endogenous redox cofactors (e.g., Coenzyme Q10)	Supports mitochondrial redox balance Reduced DNA damage in long-term rodent studies (Quiles et al., 2005)
Telomere attrition	Cycloastragenol (telomerase activation)	Increased telomerase activity (Yu et al., 2018; He et al., 2022) Telomere length increase in humans (Salvador et al., 2016) Increased healthspan and telomere length in mice without increasing cancer incidence (de Jesus et al., 2011) Improved metabolic parameters in 2-year-old mice (Yu et al., 2018) Enhanced wound healing in rats (Sevimli-Gü et al., 2011)
	Synthetic telomerase activators (e.g., AGS-499, TAC)	Increased TERT expression Enhanced neuronal survival and functional improvement in murine neurodegeneration models (Eitan et al., 2012) Reduced neuroinflammation, improved neurogenesis and cognitive performance in naturally aged mice (Shim et al., 2024)
	TERT gene therapy	Reduced critically short telomeres Improved mitochondrial and metabolic parameters without increased cancer in tested models Extended median lifespan in mice (Bernardes de Jesus et al., 2012)
Sex hormone decline (females)	Bioidentical hormone replacement therapy (HRT)	Estrogen affects all 12 hallmarks of aging (Rabinovici et al., 2025) Reduced cardiovascular risk, fracture risk, metabolic dysfunction, depression, and mortality when initiated near menopause in human females (Sullivan et al., 2016; Lobo, 2014)
Sex hormone signaling modulation (males)	Experimental non-feminizing estrogenic interventions (e.g., 17α-estradiol)	Improved insulin sensitivity, reduced inflammation and mTORC1 signaling without feminization in male mice Extended median lifespan in male mice (Stout et al., 2016; Harrison et al., 2021)

### 4.1 Targeting repair vs. damage accumulation

Oxidative stress and telomere attrition are primary contributors to the DNA damage response, which in turn drives cellular senescence, and although interventions targeting these or hormonally modulated pathways, including antioxidant supplementation (J et al., 2023; Sadowska-Bartosz and Bartosz, 2014), telomerase reactivation (de Jesus et al., 2011; Harley et al., 2013; Salvador et al., 2016), and hormone replacement therapy (HRT) (Xing Y. et al., 2023; Baik et al., 2024), have each produced benefits such as improved metabolic and cardiovascular function or delayed senescence-associated phenotypes, none have yielded substantial lifespan extension in otherwise healthy individuals (Varela-López et al., 2023; Schmidt et al., 2015). The lack of success may lie in the redundancy of the primary mechanisms, and the limited emphasis on early preventive interventions targeting redundant pathways in mammalian systems.

The major source of molecular damage is oxidative stress causing DNA damage with resulting genomic instability, as well as protein and lipid damage (da Costa et al., 2016). Findings linking DNA damage to aging suggest that enhancing nuclear DNA repair or decreasing DNA damage accumulation may facilitate slowing-down the aging process (López-Otín et al., 2013). However, targeted manipulation of repair pathways in humans presents substantial challenges (Freitas and De Magalhães, 2011). The DNA damage response involves hundreds of proteins, with ATM and ATR phosphorylating over 700 substrates following genotoxic stress, forming an extensive and highly interconnected network (Freitas and De Magalhães, 2011). In this context, upregulating a single repair factor may merely redirect the rate-limiting step elsewhere, limiting the efficacy of such interventions (Freitas and De Magalhães, 2011). Since no powerful and safe small molecules are currently approved that directly repair DNA damage or robustly and consistently enhance endogenous repair mechanisms across tissues, strategies aimed at mitigating oxidative stress and preventing molecular damage accumulation remain more viable at present.

Antioxidants were reported to be sufficient to decrease ROS and DNA damage in various cell culture models (Cervelli et al., 2017; Hardy et al., 2021; Merlin et al., 2022), and extend fibroblast replicative lifespan *in vitro* (Sadowska-Bartosz and Bartosz, 2020). *In vivo*, ataxia-telangiectasia, a condition caused by ATM deficiency, which leads to defective DNA repair and accelerated genomic instability, has been demonstrated to be influenced by antioxidant supplementation, which delayed the onset of T cell lymphoma and ameliorated neurological manifestations in ATM-deficient mice (Schubert et al., 2004; Browne et al., 2004). Additionally, antioxidant defense during inflammation is important for longevity as exemplified by the CD33rSiglec family proteins, loss of which in mice leads to oxidative imbalance, increased molecular damage, and accelerated aging (Schwarz et al., 2015). The free radical theory of aging, however, has been broadly criticized due to inconclusive and often negative results from studies in model organisms overexpressing endogenous antioxidant enzymes (Pérez et al., 2009; Buffenstein et al., 2008), as well as disappointing outcomes from antioxidant supplementation trials using compounds such as vitamin E and beta-carotene (Miller et al., 2005; Alpha-Tocopherol and Beta Carotene Cancer Prevention Study Group, 1994; Bjelakovic et al., 2007), where these molecules did not demonstrate life-extending properties and even have been linked to increased all-cause mortality. However, these findings do not necessarily invalidate the potential of antioxidants for extending healthspan and lifespan. Overexpression of antioxidant enzymes may yield compensatory responses that paradoxically disrupt redox homeostasis or fail due to redundancy in antioxidant defense pathways (Shields et al., 2021). For instance, *C. elegans* overexpressing *sod-1* exhibited elevated protein oxidation and H_2_O_2_ levels compared to wild-type (Shields et al., 2021). Human trials using antioxidants have largely relied on compounds that are either hydrophilic (e.g., vitamin C) (Bjelakovic et al., 2007), which poorly penetrate lipid-rich intracellular compartments including mitochondria, or lipophilic antioxidants like vitamin E and beta-carotene (Miller et al., 2005; Alpha-Tocopherol and Beta Carotene Cancer Prevention Study Group, 1994; Bjelakovic et al., 2007), which are metabolically active and, importantly, can act as pro-oxidants under certain conditions (Pearson et al., 2006; Vrolijk et al., 2015; Pravkin et al., 2013; Zanotto-Filho et al., 2008; Palozza et al., 2003). The pro-oxidative properties may explain increased all-cause mortality with beta carotene, vitamin A, and vitamin E supplementation (Miller et al., 2005; Alpha-Tocopherol and Beta Carotene Cancer Prevention Study Group, 1994; Bjelakovic et al., 2007).

Therefore, the exact mechanism of action and molecular properties, particularly the potential to elicit oxidative stress, must be carefully considered when selecting antioxidants for healthspan- and lifespan-extending interventions. (Vrolijk et al., 2015). Negative or inconclusive results from earlier antioxidant studies, often involving compounds with poor pharmacokinetics or off-target effects, have led to decrease in further investigation in this area, and research involving more targeted and mechanistically grounded antioxidant strategies remains limited. No large-scale randomized long-term human studies have investigated the effects on healthspan or lifespan of lipophilic, non-metabolic, nontoxic antioxidants capable of efficiently penetrating lipid and mitochondrial membranes. Antioxidants satisfying above-mentioned criteria would provide damage-prevention targeting not only DNA, but other molecules such as protein and lipids, and acting both intra- and extracellularly. While definitive data on lipophilic antioxidants other than vitamin E, A, and beta-carotene in the context of healthspan and lifespan is still limited, several compounds can be discussed due to their potency, lipophilic properties, favorable safety profile, and promising early data in model organisms and humans. Emerging evidence suggests that long-term antioxidant supplementation is sufficient to decrease oxidative stress and DNA damage in healthy mammals, and the following paragraphs outline some of the antioxidative molecules which demonstrated efficacy in mammalian systems.

Coenzyme Q10 provides initial evidence that long-term antioxidant supplementation can reduce molecular damage in mammals, as life-long administration in rats decreased DNA damage in peripheral lymphocytes (Quiles et al., 2005). However, CoQ10 is endogenous, metabolically active, and its efficacy may be restricted by limited mitochondrial accumulation with exogenous supplementation. This rationale has led to the development of mitochondria-targeted antioxidants that accumulate at the main site of reactive oxygen species production. Compounds such as SkQ1, MitoTEMPO, and XJB-5-131 reduce mitochondrial oxidative damage and avoid the limitations of endogenous antioxidants. Among these targeted approaches, Mitoquinone (MitoQ) has received the most translational attention. MitoQ is a mitochondria-targeted ubiquinone analogue that accumulates selectively within mitochondrial membranes via a lipophilic cation moiety, enabling preferential scavenging of mitochondrial reactive oxygen species. In a recent study on human umbilical vein endothelial cells treated with Doxorubicin, MitoQ co-treatment lowered mitochondrial superoxide levels, preserved mitochondrial mass, reduced DNA damage (including telomere-associated damage) and reduced senescence-associated β-galactosidase activity and senescence marker mRNA expression. These data position MitoQ as a mitochondria-targeting antioxidant candidate for preventive approaches aimed at mitigating age-related mitochondrial dysfunction and downstream cellular senescence (Abdeahad et al., 2025).

Astaxanthin is another highly potent antioxidant, which is also known to increase the expression of endogenous antioxidant enzymes (Wang et al., 2022; Monmeesil et al., 2019). Astaxanthin is a stronger antioxidant than CoQ10 (Maezawa et al., 2016), acts both inside and outside of mitochondria (Yu et al., 2019; Sun et al., 2021; Sztre et al., 2019), and has been demonstrated to decrease DNA damage measured by 8-OHdG (Park et al., 2010) and reduce skin aging in humans (Belviranlı and Okudan, 2015). Additionally, it has excellent safety profile (Brendler and Williamson, 2019), does not become pro-oxidant *in vivo* (Maezawa et al., 2016), and is not intrinsically involved in mammalian metabolic processes–compared to vitamins C, A, E, glutathione, or CoQ10 – thereby reducing off-target effects. The NIA Interventions Testing Program (ITP) reported modest lifespan extension in male mice treated with astaxanthin, even with treatment initiated at 12 months of age (Harrison et al., 2024), a time point when significant molecular damage from early development has already accumulated, potentially limiting beneficial effects.

The timing of antioxidant supplementation may be a crucial factor. To date, no studies have evaluated continuous, life-long administration of astaxanthin or mitoquinone from early life through natural death in mammalian systems. The early onset of antioxidant administration may be necessary for significant healthspan and lifespan extension based on the evidence that while methylation changes start during early embryogenesis (Gladyshev, 2020; Kerepesi and Gladyshev, 2023) as a programmed event, they continue throughout life due to damage accumulation, that DNA damage is highest in dividing cells–which are significantly more prevalent during growth and development (Kaufmann and Paules, 1996), and that early-life interventions can potentially shape aging (Bartke et al., 2022).

### 4.2 Interventions targeting telomere attrition

Telomere attrition represents another primary, redundant driver of aging, and, while human data remains limited, restoring telomerase activity may represent a complementary strategy to damage-reduction approaches.

Cycloastragenol is the most studied small molecule which activates telomerase (Yu et al., 2018; He et al., 2022). It enhances proliferation and supports the survival of neural stem cells exposed to hypoxic-ischemic injury (Meng et al., 2017). Cycloastragenol treatment elevated hepatic expression of TERT, decreased hepatic lipid accumulation, and improved glucose tolerance in 2-year-old mice (Yu et al., 2018). Published results also demonstrate increased healthspan and significant increase in telomere length in the liver with cycloastragenol treatment in C57Bl/6 mice without increasing cancer incidence (de Jesus et al., 2011). Cycloastragenol also enhanced tissue regeneration in cutaneous rat wound healing model (Sevimli-Gür et al., 2011). In humans, cycloastragenol increased telomere length in a randomized, double blind, and placebo-controlled study (Salvador et al., 2016). GRN510, a research designation for cycloastragenol, decreased fibrosis in a murine model of idiopathic pulmonary fibrosis (Le Saux et al., 2013). Several additional telomere-targeting compounds have been identified. Synthetic molecules such as AGS-499 increase TERT transcription and have demonstrated *in vivo* enhanced neuronal survival and functional improvement in murine neurodegeneration models (Eitan et al., 2012). And another small-molecule telomerase activator, TERT activator compound (TAC), increases TERT expression in murine models and human cells by reversing epigenetic repression at the TERT promoter via MEK–ERK–AP-1 signaling (Shim et al., 2024). Notably, long-term administration in mice was well tolerated without detectable carcinogenic effects while decreasing neuroinflammation and improving neurogenesis and cognitive performance in naturally aged animals (Shim et al., 2024).

Gene-delivery approaches provide an additional way of telomere restoration. In adult and old mice, a single systemic dose of AAV9-mediated TERT gene therapy increased median lifespan by approximately 24% when delivered at 12 months and by about 13% when delivered at 24 months, without increasing cancer incidence (Bernardes de Jesus et al., 2012). These benefits were accompanied by longer telomeres, reduced frequency of very short telomeres, improved mitochondrial function, enhanced metabolic indices, and lower p16 expression across several tissues (Bernardes de Jesus et al., 2012). Importantly, safety was tested in a tumor-prone context: AAV9-TERT did not accelerate initiation or progression of K-Ras-driven lung cancer, even on a p53-deficient mouse background (Muñoz-Lorente et al., 2018). Overall, *in vivo* results in murine models indicate that transient TERT expression can produce durable telomere restoration and improved physiological markers without detectable cancer promotion.

Additionally, hormonal interventions also can influence telomere length: estrogen directly upregulates hTERT transcription through estrogen receptor binding to estrogen response elements within the hTERT promoter (Kyo et al., 1999), and androgens also increase telomerase activity and TERT mRNA levels, likely via indirect mechanisms (Guo et al., 2003). For example, danazol has been successfully used in clinical trials to elongate telomeres in patients with short telomere syndromes (Townsley et al., 2016).

Telomere length decreases gradually from early adulthood onward, and accelerated telomere attrition in leukocytes is associated with increased risk of cardiovascular disease and reduced longevity (Shammas, 2011; Jäger and Walter, 2016), indicating that telomere erosion begins before overt aging phenotypes emerge and justifying preventive targeting of telomere maintenance. While telomerase-activating interventions have not demonstrated carcinogenicity *in vivo* in mammalian systems, telomerase activity is present in many human cancers, and telomerase enhancement could theoretically promote the progression of pre-existing malignancies (Jafri et al., 2016), underscoring the need for research aimed at long-term safety evaluation of telomere targeting interventions.

### 4.3 Interventions targeting hormonal profile

Although definitive lifespan studies on HRT are lacking, it is unequivocal that menopause, while especially harmful when premature, is inherently detrimental, markedly increasing the risk of death and accelerating the onset of major diseases such as cardiovascular pathology and diabetes (Ossewaarde et al., 2005; Zhu et al., 2019; Huang et al., 2023; Xing Z. et al., 2023; Asllanaj et al., 2019). Hormone replacement therapy (HRT) may represent an early geroprotective intervention in females, as estrogen influences all 12 hallmarks of aging (R et al., 2025).

In addition to alleviating menopausal symptoms and sexual dysfunction, bioidentical HRT confers multiple healthspan benefits in females (Sullivan et al., 2016). In women with premature ovarian insufficiency, bioidentical HRT is superior to oral contraceptive pills for improving bone mineral density and endothelial function, and early initiation during peri-menopause can alleviate or even induce remission of depressive symptoms (Sullivan et al., 2016). When initiated soon after menopause, HRT is protective against dementia (Sullivan et al., 2016), and if started within 10 years of menopause, it decreases fracture risk, cardiovascular disease, type 2 diabetes, and mortality (Lobo, 2014). Despite these documented benefits, current practice still emphasizes using the lowest dose for the shortest duration, and in premature ovarian insufficiency HRT is often discontinued at the average age of natural menopause (Sullivan et al., 2016).

Despite common practice among providers of denying HRT to females who are considering having children in the future, estrogen supplementation has been demonstrated to have beneficial effects on fertility (Sullivan et al., 2016), potentially extending reproductive window. While the data are lacking from large-scale trials, emerging findings suggest that estradiol supplementation may support fertility in premature ovarian insufficiency through suppression of elevated LH and FSH (Sullivan et al., 2016). These effects may improve follicular responsiveness by restoring FSH receptor sensitivity in granulosa cells and reducing aberrant luteinization (Sullivan et al., 2016). In a randomized, placebo-controlled study examining the impact of estrogen pre-treatment on ovarian response to gonadotropin stimulation in women with primary ovarian insufficiency, estradiol administration before ovulation induction led to significantly higher ovulation rates compared to placebo. Follicular development and ovulation were observed only in participants who achieved serum FSH levels of ≤15 mIU/mL, indicating that estradiol-mediated suppression of endogenous gonadotropins enhanced the ovarian response (Tartagni et al., 2007).

Furthermore, early initiation of HRT in premenopausal females based on the FSH levels or FSH/LH ratio may be beneficial since elevated FSH levels not only negatively affect fertility, but, independently from estrogen levels, are associated with multiple adverse health outcomes in humans including hypercholesterolemia (Guo et al., 2019), higher BMI and lower lean mass (Veldhuis-Vlug et al., 2021), as well as increased biomarker risk of Alzheimer’s disease (Nerattini et al., 2023). Additionally, in postmenopausal women, FSH correlates with increased bone turnover markers regardless of estradiol levels (García-Martín et al., 2012), and higher baseline FSH has been linked to greater hip fracture risk in older adults even after adjusting for estradiol, testosterone, and SHBG (Koh et al., 2024). Rodent studies further support a potential causal role: FSH has been shown to directly stimulate osteoclastogenesis and accelerate bone loss in hypogonadal mice, independent of estrogen suppression (Sun et al., 2006). Elevated FSH signaling in pancreatic islets was also demonstrated to impair glucose-stimulated insulin secretion in mice, even when estrogen levels were maintained, implicating FSH in extragonadal metabolic regulation via activating Gαi to inhibit the cAMP/PKA pathway (Cheng et al., 2023). These findings suggest that FSH may not be just a marker of reproductive aging but actively contribute to systemic dysfunction. As such, investigating the effects of early HRT initiated based on elevated FSH/LH ratios, rather than after symptomatic manifestations, may hold therapeutic relevance for preserving healthspan and mitigating aging-related decline.

The Women’s Health Initiative trials misrepresented the risks of HRT by enrolling women at a mean age of 63, roughly a decade after menopause onset, when vascular aging and morbidity have already developed (Mehta et al., 2019). This design led to a decline in hormone therapy use despite its potential benefits. Subsequent analyses and newer trials have clarified that timing is critical: current clinical recommendations support initiating hormone therapy within 5 years of menopause onset to maximize protective effects and reduce risk, particularly for cardiovascular and cognitive outcomes (Smith et al., 2011; Hodis et al., 2016). It is paramount to determine the optimal timing of initiation of HRT - whether the HRT initiation is best within 5 years from menopause as currently recommended, during clinical signs of perimenopause, or earlier when diminished ovarian reserve manifests biochemically with elevated FSH/LH ratio preceding clinical presentation. For this purpose, murine models represent a useful tool, as in 18-months-old mice the ovarian reserve is decreased by approximately 10 times compared to young mice (Hense et al., 2024), ovariectomy shortens lifespan, while ovarian transplants from young mice extend longevity, reinforcing the link between hormonal decline and aging (Hense et al., 2024).

Apart from the optimal timing of HRT initiation, HRT regimen may be an additional important consideration in the context of healthspan and lifespan requiring further investigation. While continuous combined HRT is commonly used in both clinical and preclinical settings, emerging evidence suggests that cyclic progesterone administration may confer superior neuroprotective benefits, particularly in Alzheimer’s disease models (Carroll et al., 2010).

Sex-specific hormonal decline represents a major confounding factor that is often insufficiently addressed in interventions targeting aging. Although abrupt menopause is rare in the animal kingdom, seen primarily in humans, elephants, a few whale species, and the spiny mouse, gradual depletion of ovarian reserve and loss of estrogenic signaling is a conserved hallmark of female aging across mammals. Several compounds that extend lifespan in male mice, including those tested by the Interventions Testing Program, often fail to produce comparable effects in females (Strong et al., 2025), likely due to lack of engagement with this endocrine axis. Interventions that target upstream mechanisms may not be sufficient unless combined with approaches that stabilize or restore sex hormone signaling. These sex-specific differences are important not only in supporting hormone replacement therapy as part of female healthspan-extending interventions but also in exploring the potential of selective estrogen receptor modulators or phytoestrogens for male-specific strategies. For example, even with late onset initiation, in mice the non-feminizing estrogen 17α-estradiol extended median male lifespan by up to 19 percent and 90th percentile lifespan by 7%, reduced visceral adiposity, improved insulin sensitivity, lowered inflammatory markers, and suppressed mTORC1 signaling without feminization, loss of lean mass, or cardiac dysfunction (Stout et al., 2016; Harrison et al., 2021). It would be beneficial to identify additional substances that provide protective estrogenic effects in males without inducing feminization, a side effect limiting translational application at this time.

### 4.4 Examples of complex therapeutic strategies

While early-onset preventive strategies based on targeted pharmacologic interventions against redundant primary aging mechanisms remain largely unexplored, several complex late-life experimental therapeutic approaches acting across multiple mechanistic layers of aging have recently been described.

A recent framework proposed by Lewis and de Grey (Lewis and de Grey, 2024) outlines a multi-modal rejuvenation strategy initiated in aged mice combining rapamycin (mTOR inhibition), navitoclax (BCL-2 family senolytic), telomerase gene therapy, and stem cell replacement. The authors emphasize the necessity of testing such combinatorial interventions in naturally aged rodents to better capture the complexity of late-life aging phenotypes, as opposed to relying solely on progeroid or accelerated models, since interventions tested in such models are at risk of disproportionately addressing a single cause of aging phenotype (Lewis and de Grey, 2024). While this approach remains therapeutic rather than preventive, the multi-target strategy and emphasis on the wild-type naturally aged models is an important step towards development of physiologically relevant strategies aimed at healthspan extension.

Another example of a therapeutic, late-life, multi-mechanism strategy is a recent study in which a combination of nicotinamide, α-lipoic acid, thiamine, pyridoxamine, and piperine (Gly-Low), acting through glycation-lowering and ghrelin-signaling inhibition, improved glucose homeostasis, attenuated hypothalamic aging signatures, and extended lifespan in mice (Wimer et al., 2025).

A recent murine study tested co-administration of oxytocin and an ALK5 inhibitor (A5i, a TGF-β receptor I inhibitor) initiated at 25 months of age in mice which were already frail; the combination extended median lifespan and improved healthspan only in male mice, while in middle-aged female mice this treatment demonstrated positive effects on fertility but failed to increase lifespan (Kato et al., 2025), which may be explained at least in part by the fact that primary estrogen decline was not addressed. These findings highlight the potential of hormonal interventions as part of combined strategies to improve specific dimensions of healthspan and underscore the importance of sex-specific endocrine strategies in aging research.

Finally, in the TRIIM human trial (Fahy et al., 2019), nine healthy men aged 51–65 underwent a 12-month intervention with recombinant human growth hormone, dehydroepiandrosterone, and metformin, aiming to reverse age-related thymic involution and immunosenescence. The combination yielded reductions in epigenetic age (mean ∼2.5 years), improvements in immune markers including increased naïve T cells and lymphocyte-to-monocyte ratio and decreases in CD38^+^ monocytes and CRP levels (Fahy et al., 2019). While limited by small sample size and absence of placebo control, this study exemplifies a human, late-life multi-mechanism intervention targeting endocrine, metabolic, and immune aging.

Together, these studies illustrate the feasibility of multi-component interventions in modifying late-life aging phenotypes, while underscoring the unmet need for earlier, preventive strategies that act upstream on primary mechanisms.

## 5 Mechanism-based biomarkers of aging in the context of redundant primary drivers

The development of epigenetic clocks has enabled estimation of biological age (Lu et al., 2023), however, most age-associated epigenetic changes appear to arise downstream of underlying molecular damage (Pa et al., 2016). For example, oxidative stress and chronic inflammation can influence DNA methylation patterns captured by epigenetic clocks (Pa et al., 2016). As a result, epigenetic age is a useful summary measure of cumulative physiological change, but its mechanistic resolution is limited. In this framework, aging mechanisms refer to causal biological processes that drive functional decline, whereas aging biomarkers are measurable indicators that reflect the activity or downstream consequences of those processes.

If primary mechanisms such as DNA damage, telomere attrition, and hormonal decline operate redundantly and interactively, downstream readouts alone cannot distinguish which of these upstream processes is dominant in a given individual. Two individuals with similar epigenetic age or similar senescence burden may reach those states through different combinations of oxidative DNA damage, accelerated telomere loss, or premature hormonal decline. Developing biomarkers that reflect primary mechanisms rather than only their downstream consequences is therefore important for guiding targeted interventions and for monitoring whether preventive strategies meaningfully alter upstream biology.

In this context, actionable biomarkers directly tracking primary drivers may include quantitative measures of genomic and mitochondrial DNA damage and oxidative stress, assays capturing telomere length distributions rather than average length, and hormonal profiles reflecting ovarian reserve and FSH/LH dynamics. These measures provide insight into the upstream conditions that generate downstream hallmarks. Secondary biomarkers such as epigenetic alterations, senescence burden, or mitochondrial functional markers can complement primary readouts by capturing the cellular responses to upstream injury, although their mechanistic interpretation is less direct. Tertiary features such as systemic inflammation and gut microbiome composition are valuable indicators of organismal aging burden but offer limited information about causative pathways.

Practical implementation of multi-mechanism biomarker panels presents several challenges including cost and accessibility of assays, cross-platform standardization, batch effects in high-throughput omics measurements (Leek et al., 2010), and limited portability of predictive models across cohorts with different demographic or clinical characteristics. Addressing these issues will require harmonized protocols, external validation in independent populations, and development of scalable, clinically feasible assay formats. Within this context, emerging AI and machine-learning approaches provide an opportunity to integrate these layers (Putin et al., 2016; Theodorakis et al., 2024; Wu et al., 2021). Models that incorporate mechanistic considerations and combine oxidative stress markers, telomere dynamics, DNA damage, and hormonal measurements with secondary features such as epigenetic drift may enable identification of the predominant upstream mechanisms in individual patients. Such integrative analyses may shift biomarker development toward a framework that distinguishes primary drivers from their downstream consequences, allowing more precise alignment between mechanistic interventions and the biological processes they seek to modify.

## 6 Conclusion

Aging appears to arise from a network of redundant primary drivers, among which genomic and mitochondrial DNA damage, telomere attrition, and sex hormone decline represent core upstream contributors within the hierarchical framework proposed here. These upstream processes initiate molecular and cellular decline through parallel and partly independent routes. They generate secondary changes such as cellular senescence, epigenetic alterations, disabled macroautophagy, deregulated nutrient sensing, mitochondrial dysfunction, and altered intercellular communication, which then produce tertiary outcomes including stem cell exhaustion, chronic inflammation, and dysbiosis. This proposed hierarchy represents a conceptual framework for mechanistic interpretation and guiding intervention design. Feedback loops allow downstream processes to intensify upstream lesions, which blurs mechanistic boundaries but reinforces the central role of early molecular insults. This redundancy suggests that neither single biomarkers nor single-target interventions can capture or meaningfully alter the aging process. Biomarker development therefore would benefit from focus on mechanistically anchored panels that integrate measures of oxidative stress, DNA damage, telomere dynamics, and hormonal state with downstream signatures such as epigenetic drift, senescence burden, inflammation, and gut microbiome composition. Likewise, preventive and therapeutic strategies may benefit from the coordinated, early, multi-component approaches that limit damage accumulation while preserving telomere and endocrine homeostasis. Incorporating these principles offers a path toward mechanism-aligned interventions aimed at modifying the upstream biology that drives organismal aging.
