# Correction: Mechanistic redundancy and hierarchy of aging mechanisms: implications for strategies to extend healthspan and biomarker integration

**DOI:** 10.3389/fragi.2026.1928559

**Published:** 2026-07-28

**Authors:** Maria Shvedova

**Affiliations:** BUMC Center for Aging Research, Division of Plastic and Reconstructive Surgery, Department of Surgery, Boston University School of Medicine, Boston, MA, United States

**Keywords:** aging, biomarkers, DNA damage, estrogen, menopause, oxidative stress, preventive interventions, telomere attrition

There was a mistake in Table 1 as published. All references in Table 1 are wrong. The corrected Table 1 appears below.

**TABLE 1:** Interventions targeting hypothesized primary aging mechanisms.

Target	Intervention	Proposed mechanisms and published outcomes
Damage accumulation	Exogenous lipophilic antioxidants (e.g., astaxanthin)	Improves mitochondrial function (Yu et al., 2019; Sun et al., 2021; Sztretye et al., 2019) Non-pro-oxidant profile (Maezawa et al., 2016) Increases expression of endogenous antioxidant enzymes (Wang et al., 2022; Monmeesil et al., 2019) Decreased oxidative DNA damage (Park et al., 2010) and reduced skin aging in humans (Belviranl et al., 2015) Lifespan extension in male mice in ITP (Harrison et al., 2024)
	Mitochondria-targeting antioxidants (e.g., MitoQ, SkQ1, MitoTEMPO, and XJB-5-131)	Selective accumulation in mitochondria Reduction of mitochondrial ROS Decreased DNA damage and cellular senescence markers in preclinical models (Abdeahad et al., 2025)
	Endogenous redox cofactors (e.g., Coenzyme Q10)	Supports mitochondrial redox balance Reduced DNA damage in long-term rodent studies (Quiles et al., 2005)
Telomere attrition	Cycloastragenol (telomerase activation)	Increased telomerase activity (Yu et al., 2018; He et al., 2022) Telomere length increase in humans (Salvador et al., 2016) Increased healthspan and telomere length in mice without increasing cancer incidence (de Jesus et al., 2011) Improved metabolic parameters in 2-year-old mice (Yu et al., 2018) Enhanced wound healing in rats (Sevimli-Gü et al., 2011)
	Synthetic telomerase activators (e.g., AGS-499, TAC)	Increased TERT expression Enhanced neuronal survival and functional improvement in murine neurodegeneration models (Eitan et al., 2012) Reduced neuroinflammation, improved neurogenesis and cognitive performance in naturally aged mice (Shim et al., 2024)
	TERT gene therapy	Reduced critically short telomeres Improved mitochondrial and metabolic parameters without increased cancer in tested models Extended median lifespan in mice (Bernardes de Jesus et al., 2012)
Sex hormone decline (females)	Bioidentical hormone replacement therapy (HRT)	Estrogen affects all 12 hallmarks of aging (Rabinovici et al., 2025) Reduced cardiovascular risk, fracture risk, metabolic dysfunction, depression, and mortality when initiated near menopause in human females (Sullivan et al., 2016; Lobo, 2014)
Sex hormone signaling modulation (males)	Experimental non-feminizing estrogenic interventions (e.g., 17α-estradiol)	Improved insulin sensitivity, reduced inflammation and mTORC1 signaling without feminization in male mice Extended median lifespan in male mice (Stout et al., 2016; Harrison et al., 2021)

The original version of this article has been updated.

